# Improving well-being and enhancing awareness in patients undergoing hemodialysis through the person-centered IARA model: an exploratory study

**DOI:** 10.3389/fmed.2024.1425921

**Published:** 2024-07-01

**Authors:** Sara Di Marco, Anna M. Padovan, Novella Conti, Francesca Aimasso, Francesca Viazzi, Vincenzo Fontana, Dalila Campanella, Goran Kuvačić, Andrea De Giorgio

**Affiliations:** ^1^Nephrology, Dialysis, and Transplant Unit, IRCCS Ospedale Policlinico San Martino, Genova, Italy; ^2^Associazione Kiara, Rome, Italy; ^3^Clinical Epidemiology Unit, IRCCS Ospedale Policlinico San Martino, Genova, Italy; ^4^Faculty of Kinesiology, University of Split, Split, Croatia; ^5^Faculty of Psychology, eCampus University, Milan, Italy; ^6^Klinikos Center for Psychodiagnostics and Psychotherapy, Rome, Italy

**Keywords:** psychological state, person-focused, patient-centered, adherence to care, chronic hemodialysis

## Abstract

Chronic kidney disease (CKD) globally represents a significant health challenge, particularly among patients undergoing chronic hemodialysis. A careful nutritional and pharmacological prescription plays a key role in the effective management of these patients to optimize serum electrolytes, such as potassium, phosphorus, and protein intake. Furthermore, these patients can suffer psychological distress due to dietary restrictions and tight medication schedules. The present study explores the effectiveness of the person-centered IARA model in improving physiological markers and quality of life in CKD patients undergoing hemodialysis treatment. To demonstrate the effectiveness of the IARA model, 60 patients (*M* = 40; *F* = 20; 60.5 ± 9.9 years) undergoing thrice-weekly hemodialysis sessions were enrolled and randomly and blindly assigned to the Control or IARA group. The reduction in abnormal blood potassium, phosphorus, and total protein levels was investigated, alongside the psychological state through the SF-12 questionnaire. Preliminary findings showed a discernible reduction in the frequency of abnormal blood K (> 5.0 mmol/L) and P (> 4.5 mmol/L) levels in the IARA group compared to the Control group. In particular, such reductions were approximately 40% for K (OR = 0.57; 95% CL = 0.23/1.46) and about 15% for P (OR = 0.86; 95% CL = 0.27/2.74). A similar tendency was also observed for patient fluid intake during each hemodialysis session, with the frequency of higher-risk patients in the IARA group being 50% lower (OR = 0.50; 95% CL = 0.07/3.79) than that of the Control group. Although preliminary findings from this study suggest that the IARA model may have a positive effect on CKD patients’ subjective wellbeing and quality of life (QoL), further research is needed to understand the long-term impact of the IARA intervention.

## 1 Introduction

Chronic kidney disease (CKD) defined as the presence of kidney damage that persists for more than three months, is among the most prevalent chronic degenerative diseases in the world (1, 2). CKD has become increasingly prevalent also in developing nations, with a rising number of patients requiring hemodialysis treatment (1, 3). Data collected from the Italian Registry of Dialysis and Transplantation (RIDT), linked to the “Società Italiana di Nefrologia” highlight that in Italy there are nearly 50,000 men, women, and children on dialysis, about 10,000 new dialysis admissions on average in the past years, with 7,000 patients on the waiting list for a kidney transplant [RIDT (4)]. Patients often do not realize that they have kidney disease, as it is largely asymptomatic until the kidney function is severely compromised. People with CKD, and even more people in chronic hemodialysis (pHD), present significant and possibly life-threatening alterations in potassium (K), phosphorus (P), and albumin (5–7). Both hypokalemia and hyperkalemia have been associated with increased mortality in people on maintenance hemodialysis (8) with a significant risk for cardiac arrhythmias during both conditions (9). Chronic dialysis, usually, through intermittent treatments delivered three times a week, can restore K balance, but large fluctuations in serum K concentration between dialysis sessions are common. Therefore, comprehensive strategies aimed at improving the nutritional status of such patients should also address the correction of serum K levels (8).

Hyperphosphatemia is usually asymptomatic and it is widely associated with a major cardiovascular risk and increased mortality in pCKD and people undergoing hemodialysis (10). Dietary phosphate restriction and the use of phosphate binders are still considered the most effective strategies for the prevention of vascular calcification in pCKD/pHD (11). Alterations in bone metabolism are very common in pCKD/pHD and are often associated with increased mortality and morbidity. Furthermore, pCKD/pHD have a higher frequency of malnutrition than the general population (12, 13); protein intake should be increased following the initiation of dialysis to improve their survival (14, 15). Poor adherence to the treatment regimen becomes a major issue in this population, contributing to increased morbidity and mortality. The Foundation Kidney Disease Outcome Quality Initiative (16) and the Kidney Disease Improving Global Outcomes (17), recommend that pCKD/pHD receive dietary counseling and be followed up and regularly evaluated as part of a therapeutic and dietary education program. Therefore, healthcare professionals (HCp), patients, and caregivers require constant education about the benefits of dietary and therapeutic recommendations versus the risks of nonadherence (18–20). One of the models outlined in the literature that proves beneficial in enhancing both patients’ quality of life and adherence to treatment is the person-centered IARA model. IARA is an Italian acronym—Incontro, Alleanza, Responsabilità, Autonomia, i.e., Meeting, Compliance, Responsibility, Autonomy and it is already used on chronic tension-type headache (21), COPD (22); GERD (23) with encouraging results particularly toward emotion management (24). This approach encourages the natural process of personal development in a maieutic way, that is, by bringing out from the person himself some useful solutions, through practical techniques such as meditation, guided imagery, and active listening by healthcare professionals aiming to empower individuals to take responsibility and maximize their autonomy in managing the illness (25, 26). IARA has thus widely demonstrated effectiveness in improving treatment adherence, achieving higher levels of awareness about their disease (27–29) and significantly improving their quality of life, even in chronic diseases (22, 23). In light of the foregoing, IARA fits into the groove of the so-called medical humanities (30). In this preliminary investigation, through a longitudinal study based on repeated measurements, we aimed to explore the effectiveness of the IARA model in improving physiological markers and quality of life in pGKD undergoing dialysis treatment at the Nephrology, Dialysis, and Transplant Unit of the IRCCS Policlinico San Martino, Genoa, Italy.

## 2 Materials and methods

### 2.1 Participants

The study was approved by the Institutional Ethical Committee of the IRCCS Policlinico San Martino and written consent was obtained by all participants. Sample size calculations defined a cohort of 60 patients to be selected based on their medical history from all pHD followed up at the Nephrology Dialysis and Transplant Unit.

Eligibility criteria for participation included being a native Italian speaker, undergoing thrice-weekly dialysis sessions, not being residents in nursing homes (RSA), not having a clinical diagnosis of dementia, or stage 5 chronic kidney disease according to K/DOQI classification, and being aged between 25 and 75 years.

**Figure 1:**
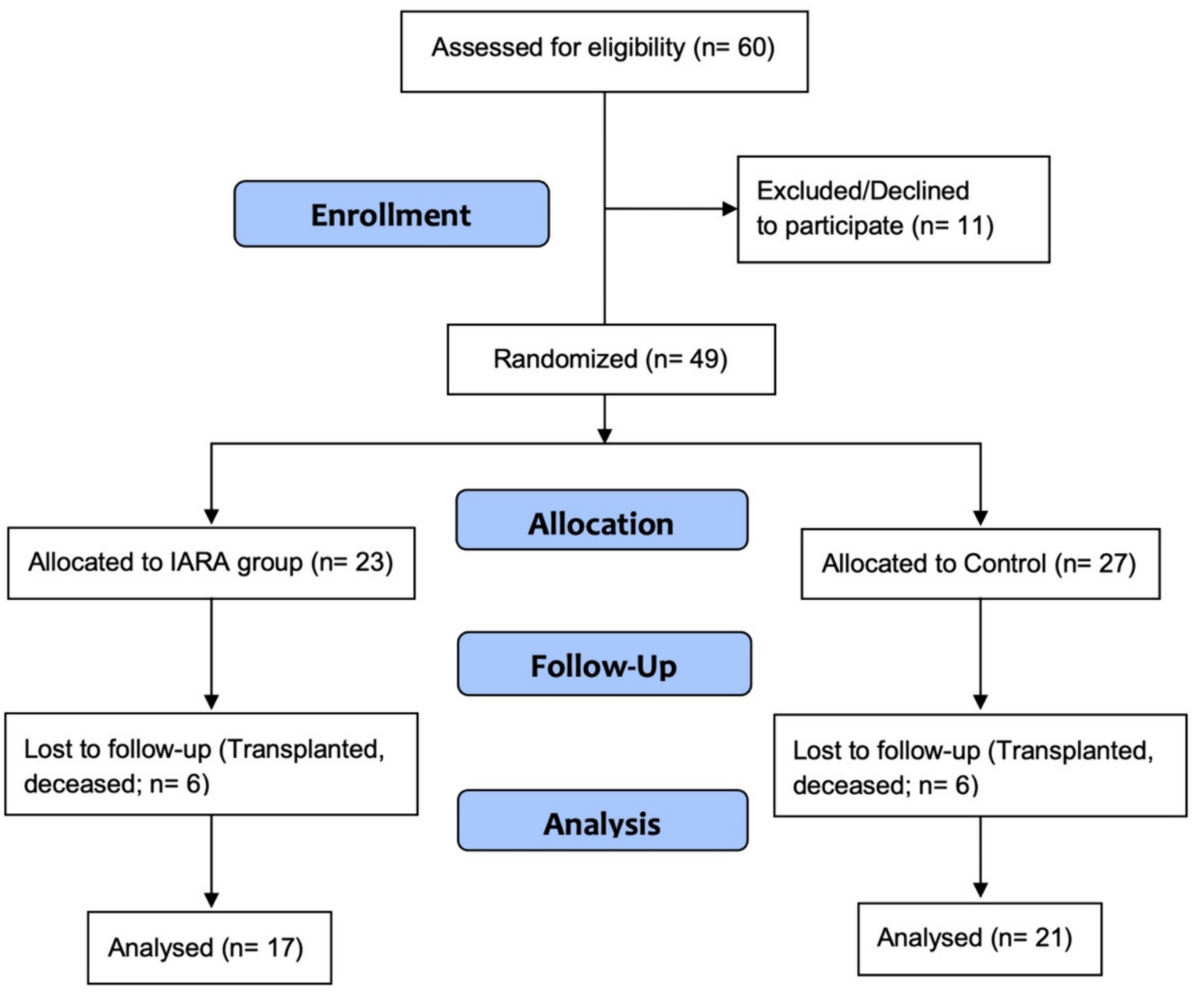
Consort diagram. The final sample, excluding dropouts, was composed of 17 participants for IARAg and 21 for HDg.

Patients were selected according to a two-step procedure (Figure 1) by using a software-based random number generator. Firstly, 60 out of all (about 150) eligible patients followed up at the Nephrology Dialysis and Transplant Unit were gathered using a simple random sampling technique. Among them, there were 40 males and 20 females with an overall mean age of 60.5 ± 9.9 years. Secondly, a random binary (0/1) indicator was created to blindly assign the 60 sampled patients to control (HDg; 18 males and 9 females; mean age = 62.1 ± 7.2 years) or experimental (IARAg; 22 males and 11 females; mean age = 59.1 ± 11.6 years) group. A written consent was obtained from all patients.

### 2.2 Phases of intervention, blood sampling, and weight assessment

The initial data collection and informed consent process took place 10 days before the first meeting. In general, IARAg had a 2-h session led by a case management nurse with and supervised by a clinical psychologist, both trained in the IARA model, while the HDg continued with standard healthcare.

The standard treatment provided by the Hemodialysis Unit includes a visit from a Nephrologist at each dialysis session and the continuous presence of a dedicated nurse during the treatment, adhering to the WHO-recommended ratio of 1:3.2. Upon admission to the Hemodialysis Unit, patients receive informational brochures and standardized diets, which are developed collaboratively by the medical and dietitian teams. These diets are tailored to the patient’s caloric intake and any concurrent conditions. Finally, if requested by either the nephrologist or the patient, a Dietitian and a Psychologist are available at the IRCCS San Martino Polyclinic Hospital.

The IARA meetings were conducted as follows. The first meeting (T0) involved assessing the participant’s understanding of their condition, identifying personal strengths, by drawing the “awareness drawing” which led to exploring solutions to physical discomfort [see, e.g., (22, 25, 27)]. Moreover, the nurse provided educational materials which included nutritional indications. The education material included videos performed by nephrologists and dietitians explaining both the pathology and diet.

The second meeting (T1) occurred 20 days after the previous one (HDg continued with standard healthcare), focusing on sharing experiences, addressing doubts about educational materials, and discussing both nutritional aspects and pathology. The nutritional aspects are explored more comprehensively. The nurse delved into the problems regarding K and P levels, water accumulation, and water restriction, explaining what happens at an organic level and what risks there are with the accumulation of both electrolytes and liquids. Moreover, participants shared their experiences, identified personal strengths, and engaged in imagery exercises tailored for them following the IARA model (23), visualizing hemodialysis therapy to improve awareness, creating a state of serenity, and improve the wellbeing.

The third meeting (T2) occurred 20 days after T1 (HDg continued with standard healthcare), still focusing on sharing experiences, recalling identified personal strengths, and engaging in the same imagery exercise.

Follow-up assessments for both groups were conducted at 3, 6, and 12 months from T2 in order to evaluate learning outcomes and overall health status, providing support to the individuals through phone consultations by the nurse case management. The body weight is also assessed evaluating the risk index (WIMR – dichotomous indicator) through the theoretical weight (estimate of the patient’s body weight carried out by the nephrologist), the initial body weight of the dialysis session, maximum hourly weight loss (estimate of the hourly weight loss carried out by the nephrologist), and hours of dialysis treatment. Then, WIMR = 0 was set if the patient falls within the theoretical body weight; otherwise, we set WIMR = 1.

To investigate the psychological and physical factors, the SF-12 questionnaire was administered. SF-12 is based on a subset of 12 items derived from the SF-36 (31). The main outcome measures include physical composite score and psychological composite score as determined by the SF-36 and SF-12 (32). Originally developed from the SF-36, the SF-12 has considerable accuracy and yet far less respondent burden.

Finally, because biological indicators (K, P, albumin, and total protein) can be measured with the range of normal physiological values, we decided to study them as binary values, i.e., below (= 0) or above (= 1) desirable values.

### 2.3 Statistical analysis

The distributions of demographic, clinical, and psychophysical characteristics were explored using descriptive statistics.

The baseline distributions of all metric variables (i.e., age at recruitment, blood levels of K, P, total proteins and albumin; pre−/post-dialysis and dry body weights) were expressed in terms of mean, median (P50), standard deviation (SD), interquartile range (IQR) and range of variation (min-max). In this context, the Student’s *t*-test for two independent samples was applied to compare the IARAg and HDg mean values of all variables. The analysis of contingency tables was used to describe the joint distributions of all baseline dichotomous variables (i.e., gender, WIMR, physical and mental status), and the treatment group indicator (IARAg vs. HDg) and the chi-squared test was performed to assess the association between two dichotomous factors. Mixed effects logistic regression modeling (LRM) (33) was used to estimate the effect of IARA intervention separately on each primary (K, P, albumin, total proteins, WIMR) and secondary (physical and psychological status) endpoint. For this reason, as already stated, all blood parameters and psychological features were split into two categories, the former according to the upper limits of the normality ranges (K > 5.0 mmol, *p* > 4.5 mmol, albumin >50 g/L, total proteins >82 g/L) and the latter following the median values (physical status score > 33; psychological status score > 44). As regards WIMR, patients were divided into higher (WIMR = 1.0) and intermediate lower (WIMR <1.0) risk groups.

In LRM, the fixed effect was represented by the treatment group indicator (IARAg vs. HDg) while the random effect was represented by the measurements repeated on each patient. The odds ratio (OR), along with the corresponding 95% confidence limits (95%CL), was computed as an index of association adjusted for age at recruitment, gender, and baseline measurements of each clinical and psychological parameter. LRM was also used for a comparative evaluation of the IARA intervention at 3 and 6 months and at the end of the study (12 months). A *p*-value of ≤0.05 was considered statistically significant. All statistical analyses were performed using STATA software (StataCorp. Stata: Release 17. Statistical Software. College Station, TX: StataCorp LP, 2021).

## 3 Results

As shown in Figure 1, 11 (18.3%) out of the 60 patients recruited in the study refused to participate, 7 (11.7%) of whom were allocated to HDg and 4 (6.6%) to IARAg (Supplementary Table S2). In addition, another 12 patients withdrew early from the observation because of transplant (4), death (7), or dropout (1), 6 belonging to HDg and 6 to IARAg (Supplementary Table S3). Tables 1, 2 report, respectively, the statistical description of metric and binary variables at baseline of the 49 patients available for analysis. By and large, no significant mean differences were found between the two treatment groups although remarkable discrepancies were pointed out for age at recruitment (62.3 vs. 56.9; *p* = 0.071) and blood K levels (5.34 vs. 6.17; *p* = 0.051; Table 1).

**Table 1:** Distribution of metric variables measured at baseline in the control (HDg) and experimental (IARAg) groups.

Variable	HDg (*N* = 27)					IARAg (*N* = 22)					*P*-value
	Mean	SD	P50	IQR	Min-Max	Mean	SD	P50	IQR	Min-Max	
Age at recruitment	62.3	7.15	62.3	8.76	46.1–75.1	56.9	13.1	57.2	20.2	35.7–75.6	0.071
Years of dialysis	4.59	3.63	3.97	3.56	1.22–16.0	5.42	6.96	2.91	1.94	0.40–24.0	0.714
Phosphorus (P)	5.34	0.60	5.40	0.70	4.20–6.50	5.53	0.58	5.60	0.80	4.10–6.30	0.276
Potassium (K)	5.24	1.61	5.50	2.70	2.30–8.40	6.17	1.64	5.85	2.60	3.50–9.10	0.051
Total proteins	65.3	4.38	65.6	5.60	54.5–72.5	66.6	6.33	66.7	6.50	54.1–79.3	0.384
Albumin	38.8	2.90	38.7	3.90	32.6–44.1	39.0	3.85	39.2	5.80	32.6–47.1	0.869
Pre-dialysis weight	77.0	26.8	69.2	30.9	49.5–140.5	72.0	15.0	73.3	24.2	41.5–91.3	0.441
Post-dialysis weight	74.7	26.2	67.0	31.4	47.1–135.8	70.0	14.6	71.9	26.0	40.0–88.0	0.454

N, sample size; SD standard deviation; P50, median; IQR, inter-quartile range; *P*-value, probability level associated with Student’s *t*-test.

**Table 2:** Distributions of binary characteristics evaluated at baseline in the control (HDg) and experimental (IARAg) groups.

Variables and categories	HDg (*N* = 27)		IARAg (*N* = 22)		*P*-value
	*n*	%	*n*	%	
Gender					0.647
Male	18	66.7	16	72.7	
Female	9	33.3	6	27.3	
Weight increases mean risk					0.966
<1.00	21	87.8	15	87.3	
=1.00	6	22.2	7	22.7	
Physical status classification score					0.308
Lower (0–33)	15	55.6	9	40.91	
Higher (34–56)	12	44.4	13	59.09	
The psychological status classification score					0.851
Lower (0–44)	14	51.9	12	54.55	
Higher (45–65)	13	48.1	10	45.45	

N, sample size; n/%, absolute/relative frequency; *P*-value, probability level associated with the chi-squared test.

Furthermore, the data are not modified based on the type of dialysis treatment or the type of vascular access because the HDg and IARAg data are equivalent (Table 3).

**Table 3:** Evaluation of the type of vascular access and the type of dialysis treatment.

VARIABLE	HDg	IARAg
AVF	21 (58.33)	15 (41.67)
CVC	6 (46.15)	7 (53.85)
Test X^2^ (*p*-value) = 0.523 (0.449)		
HDF	18 (52.94)	16 (47.06)
HD	6 (60.00)	4 (40.00)
AFB	3 (60.00)	2 (40.00)	HF	.	.
Test X^2^ (*p*-value) = 0.210 (0.900)

AVF, Arteriovenous fistula; CVC, Central Venous Catheters; HDF, hemodiafiltration; HD, hemodialysis; AFB, acetate-free biofiltration; HF, hemofiltration.

The results of the LRM analysis are shown in Table 4.

**Table 4:** Effect of treatment group (IARAg vs. HDg) on clinical and psychological parameters and related time trend over the follow-up period estimated through a mixed-effects logistic regression approach.

Variables and categories	Potassium levels > 5.0 vs ≤ 5.0 mmol/L			Phosphorus levels > 4.5 vs ≤ 4.5 mmol/L			Weight mean increase risk = 1 vs < 1			Physical status classification Higher vs lower score			Psychological status classification Higher vs lower score		
	OR	95%CL	*P*-value	OR	95%CL	*P*-value	OR	95%CL	*P*-value	OR	95%CL	*P*-value	OR	95%CL	*P*-value
Treatment group			0.242			0.799			0.503			0.828			0.125
HDg	1.00	(Ref.)		1.00	(Ref.)		1.00	(Ref.)		1.00	(Ref.)		1.00	(Ref.)	
IARAg	0.57	0.23/1.46		0.86	0.27/2.74		0.50	0.07/3.79		0.88	0.27–2.89		1.89	0.84–4.29	
Time at examination			0.920			0.689			0.285			0.891			0.511
3 mo	1.00	(Ref.)		1.00	(Ref.)		1.00	(Ref.)		1.00	(Ref.)		1.00	(Ref.)	
6 mo	0.97	0.38/2.47		1.41	0.46/4.16		0.29	0.06/1.51		0.98	0.41–2.36		0.69	0.29–1.62	
12 mo	0.82	0.31/2.19		0.87	0.29/2.57		0.45	0.09/2.38		0.82	0.33–2.04		0.61	0.25–1.50	

OR, odds ratio adjusted for age at recruitment, gender, and baseline measurements of each clinical and psychological parameter; 95%CL, 95% confidence limits for OR; *P*-value, probability level associated with the likelihood ratio test; Ref., reference category.

It is worth noting discernible reductions in the frequency of abnormal blood K (> 5.0 mmol/L) and P (> 4.5 mmol/L) levels in IARAg when compared to HDg. In particular, such reductions were, respectively, of about 40% for K (OR = 0.57; 95%CL = 0.23/1.46) and about 15% for P (OR = 0.86; 95%CL = 0.27/2.74).

A similar tendency was also estimated for WIMR with a frequency in IARAg of patients at higher risk which was 50% (OR = 0.50; 95%CL = 0.07/3.79) lower than that of HDg.

As far as the two psychological features are considered, discrepant findings were instead obtained. Specifically, the proportion of patients with a higher physical status score (> 33) among IARAg patients was about 10% (OR = 0.88; 95%CL = 0.27–2.89) lower in comparison to HDg patients. By contrast, using the same referent, an excess of about 90% (OR = 1.89; 95%CL = 0.84–4.29) of patients with a higher psychological status score (> 44) was found in IARAg.

Albumin and total protein levels were not considered in the LRM analysis because no abnormal measurements (albumin >50 g/L; total proteins >82 g/L) were found over the entire follow-up period.

It is noteworthy a generalized decreasing tendency of all outcomes in particular at the last examination (12 months) when compared to the first (3 months). Similar findings were also pointed out for the physical and psychological status classification scores (Table 4).

## 4 Discussion

Although no statistically significant result was obtained from LRM analysis, allegedly due to the refusals and losses of patients over the study period, some important findings were pointed out and deserve to be carefully considered. The present investigation can be classified as an underpowered study, essentially because of the recruited patients who refused to participate. However, the comparisons between participants and non-participants in terms of baseline characteristics (Supplementary Table S2) did not seem to point out discernible differences, testifying to some extent the absence of a selective (non-compliance) refusal. Also, losses to follow-up due to transplant, death, and dropout appeared to be distributed evenly between the two groups (Supplementary Table S3).

The findings from the analysis conducted in this study shed light on the potential effectiveness of the IARA model in improving both physiological and psychological outcomes in patients undergoing dialysis treatment. Firstly, the results indicate notable reductions in the frequency of abnormal blood potassium and phosphorus levels among IARAg compared to HDg. It is crucial to emphasize that monitoring electrolyte imbalances is crucial even in the general population (34), suggesting that even mild electrolyte disorders can have detrimental effects. The reductions observed in our study concerning abnormal K and P levels in the IARAg when compared to the HDg, were around 40 and 15%, respectively. These differences highlight how the IARA model may contribute to better management of electrolyte imbalances in this population, which is crucial for their overall health and wellbeing. Moreover, WIMR in the IARAg demonstrated a 50% lower frequency of higher-risk patients compared to those in the HDg. The graphical representations of the time trends further support the observed improvements in physiological outcomes, with a general decreasing tendency in abnormal K and P levels and WIMR over the follow-up period. This indicates a potential sustained benefit of the IARA intervention in managing biochemical parameters among dialysis patients, suggesting that IARA may help in reducing the occurrence of complications and adverse events associated with dialysis treatment. Adherence to therapy in patients with chronic kidney disease is crucial (19, 20) and can affect both biochemical and psychological parameters. The latter, in our case, presents a more nuanced picture. While there was a slight decrease in the proportion of IARAg with higher physical status scores compared to HDg, indicating a potential worsening in physical wellbeing, the opposite trend was observed for psychological status scores. It is possible to infer that the IARA model increased awareness of one’s illness in the experimental group, as already demonstrated (22, 25), leading to improved psychological indices. This inference was somewhat suggested by an IARAg patient who stated: “After so many years of dialysis and suffering, I have now come to know my potential and latent qualities. This helped me to improve my diet and I accepted my illness as something to make peace with rather than fight it.” It cannot be excluded that early use of IARA may show even greater differences between an experimental and a control group, also if it is important to highlight how many other factors can contribute to the wellbeing of hemodialysis patients. Cukor et al. (35) discussed how psychosocial aspects such as psychopathology, social support, family issues, and socioeconomic status can affect the wellbeing of this population, also emphasized that end-stage renal disease patients who receive continuous monitoring and support from HCp may represent a promising target population for implementing interventions aimed at lowering morbidity and mortality rates. More recently, Battaglia et al. (36) highlighted, through an interesting preliminary study, how important it is to assess the psychosocial dimensions in hemodialysis patients on the transplant waiting list, dimensions that can contribute to the wellbeing of these people who remain waiting, at least in Italy, for a long time, an average of 3.2 years (36).

As already written, one of the most contributing factors to increased morbidity and mortality in pHD is poor adherence to the treatment regimen. One of the main elements promoted by IARA is precisely the adherence [to care] promoted by a trusting relationship with HCp. Indeed, a good relationship with physicians and nurses, characterized by empathy, active listening and support, increases the patient’s confidence in treatment (37). The first objective of IARA, explicated by the *meeting*, is precisely to create a climate of empathy in which the health worker uses active, open, non-judgmental, and welcoming listening toward the patient. In this way, the patient can experience a relationship of trust that will lead him in turn to be welcoming toward the requests of the HCp. The IARA was also promoted as a training for HCp (38) in order to enhance communication. Indeed, it has been demonstrated that the main causes of non-adherence include the chronic nature of the disease, denial of the disease accompanied by fear and avoidance behaviors, perceived communication barriers with medical staff, and lack of information or difficulty remembering it (39). More recently, El-Magd et al. (18) explored the association between symptoms of anxiety and depression with dialysis withdrawal. The research revealed that anxiety and depressive symptoms at baseline were linked to a higher likelihood of dialysis withdrawal, independent of somatic comorbidities. Patients with more severe anxiety and depressive symptoms were found to be more vulnerable to dialysis withdrawal, indicating the importance of understanding factors influencing this decision-making process. Process. It can therefore be inferred that the patients, already fatigued by dialysis and perhaps particularly anxious or depressed, refused to participate in a study that would have required them to prolong their hospital stay. This point highlights that for a future exploration of the effectiveness of the IARA model on this particular category of patients, it will be useful to investigate their anxiety and depression indices beforehand. Added to this is the fact that the loss of approximately 20% of participants in the follow-up period has undoubtedly compromised the statistical power, thereby preventing even substantial differences between the two groups from reaching statistical significance. It is therefore conceivable that recruiting non-depressed or anxious patients beforehand could result in increased initial participation as well as a decrease in the number of dropouts during the study period. With a larger sample, the results would have been similar and, at least in some cases, potentially significant, a trend that also seems to apply to losses in the follow-up period.

The findings of this study provide preliminary evidence for the effectiveness of the IARA model in improving both physiological and psychological outcomes in patients undergoing dialysis treatment.

Despite the encouraging trend, further studies are needed to define the full efficacy of IARA in hemodialysis. These studies should take into account: (i) the use of a more representative sample; (ii) the psychological and social assessment when taking charge of the patient; (iii) the patients’ perception of the communication they had with the HCp; (iv) the degree of empathy they feel for their relationship with HCp. The last two suggestions would find full expression within qualitative research.

## 5 Limitations of the study

We acknowledge that the described study is not generalizable or statistically significant due to the small sample size. However, the results obtained indicate a trend that warrants further investigation with an expanded sample. In particular, we collected testimonies from patients regarding the subjective effectiveness of the IARA model on them. For this reason, a qualitative analysis could highlight the most significant results concerning the psychological wellbeing of this particular category of patients.

## Data availability statement

The original contributions presented in the study are included in the article/Supplementary material, further inquiries can be directed to the corresponding author.

## Ethics statement

The study was approved by the Institutional Ethical Committee of the IRCCS Policlinico San Martino. The studies were conducted in accordance with the local legislation and institutional requirements. The human samples used in this study were acquired from a by- product of routine care or industry. Written informed consent for participation was not required from the participants or the participants’ legal guardians/next of kin in accordance with the national legislation and institutional requirements.

## Author contributions

SM: Conceptualization, Data curation, Funding acquisition, Investigation, Methodology, Validation, Visualization, Writing – original draft, Writing – review & editing. AP: Conceptualization, Investigation, Methodology, Writing – original draft, Writing – review & editing. NC: Validation, Visualization, Writing – original draft, Writing – review & editing. FA: Validation, Visualization, Writing – original draft, Writing – review & editing. FV: Supervision, Validation, Visualization, Writing – original draft, Writing – review & editing. VF: Formal analysis, Investigation, Methodology, Writing – original draft, Writing – review & editing. DC: Formal analysis, Investigation, Methodology, Writing – original draft, Writing – review & editing. GK: Methodology, Supervision, Validation, Visualization, Writing – original draft, Writing – review & editing. ADG: Conceptualization, Data curation, Formal analysis, Funding acquisition, Investigation, Methodology, Project administration, Resources, Supervision, Validation, Visualization, Writing – original draft, Writing – review & editing.
