# Supplementary material for: Improving well-being and enhancing awareness in patients undergoing hemodialysis through the person-centered IARA model: an exploratory study
Source: Front Med (Lausanne). 2024 Jul 1;11:1425921. doi: 10.3389/fmed.2024.1425921 (PMC11247009; doi:10.3389/fmed.2024.1425921)
Supplement: Supplementary file 1 [file Data_Sheet_1.PDF]

Supplementary Materials

1. Sample size calculations

In order to determine the number of participants to be recruited in the study, one of the primary endpoints was utilized. Given the high statistical correlation between blood concentrations of phosphorus and potassium, the latter electrolyte was chosen as the reference index. Table S1 and Figure S1 present the data of 95 pCKD followed up at the Nephrology, Dialysis, and Transplant Unit of the IRCCS Ospedale Policlinico and categorized based on their clinical condition, defined as less severe (medium-low severity) and more severe (medium-high severity).

Clinical severity	*N*	*Mean*	*SD*	*NI*-95%	*Median*	*Range*
Low-medium	38	4.4	0.387	3.7-5.2	4.5	3.4-5.0
Medium-high	57	5.7	0.581	4.5-6.8	5.6	3.3-7.5
Total	95	5.2	0.786	3.6-6.7	5.3	3.3-7.5

Table S1 - Distribution of observed potassium concentration in 95 hemodialyzed patients divided by severity of clinical condition. Legend - N: sample size; M: mean; SD: standard deviation; NI-95%: range containing 95% of observed measurements; Range: minimum and maximum value.

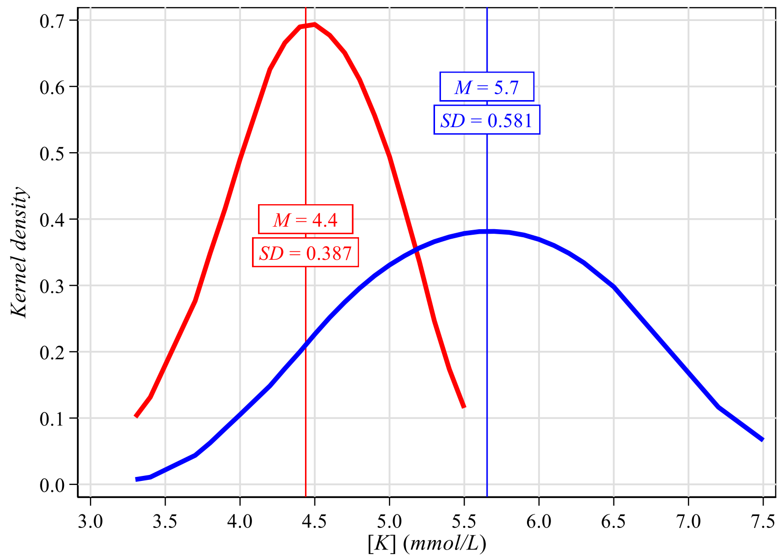

Figure S1 - Kernel density distributions of observed potassium concentrations ([K]) in 95 hemodialyzed patients, including 38 with a medium-low severity clinical condition (red curve) and 57 with medium-high severity. Legend - M: mean; SD: standard deviation.

Considering the normal limits (NL) of K levels (3.5-5.0 mmol/L), it is observed (Table S1) that individuals with lower severity show an unaltered clinical picture, with a mean value and a 95% interval (CI-95%) within the NL. Conversely, individuals with higher severity show markedly aberrant indices, indicative of a clear functional impairment.

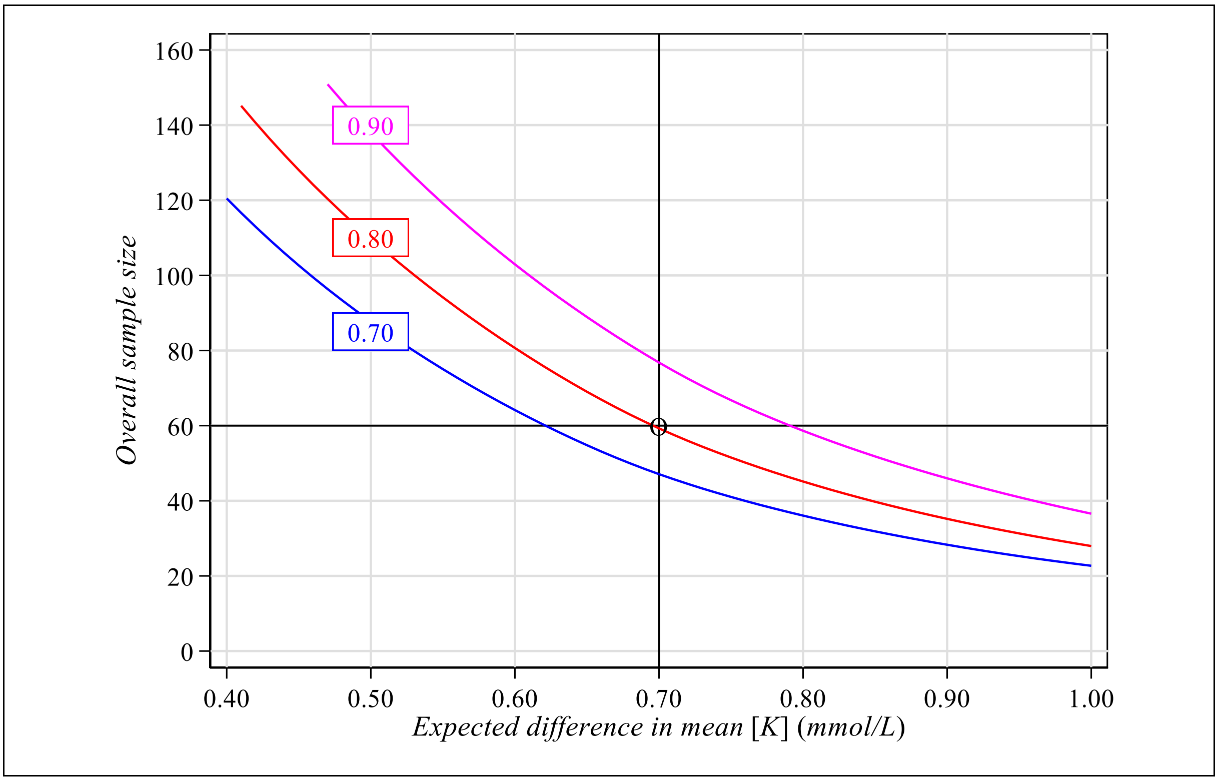

Figure S2. Estimation of sample size as a function of the expected difference in potassium blood levels between IARAg and HDp for three values of statistical power (0.70, 0.80, and 0.90).

Assuming that at the end of the first follow-up period (12 months), patients in both IARAg and HDg have K levels similar to those of individuals with lower and higher clinical severity, it is possible to identify a precise expected mean difference attributable to the IARA intervention of 0.7 mmol/L, a minimum value necessary for an assisted individual to fall within the NL (5.7-0.7 = 5.0). Therefore, with a type I error of 0.05 (two-tailed test), a type II error of 0.10 (power = 0.90), a common standard deviation (SD) of 0.9 mmol/L, and a 1:1 ratio of patients in IARAg to patients in HDg, the total sample size required for the minimum expected difference in potassium blood levels of 0.70 mmol/L to be statistically significant (p-value ≤ 0.05) must be approximately 54 patients, 27 per group. Considering a dropout rate of 10%, a total of 60 assisted individuals will be needed, 30 per group (Figure 2S).

Variables and categories	*Accepted*		*Refused*		*P-value*
	N	%	*N*	%	
Treatment group					0.606
HDg	27	55.1	7	63.6	
IARAg	22	44.9	4	36.4	
Years since first dialysis (*M*) (*SD*)	(4.7)	(5.3)	(4.6)	(3.7)	0.719
0.2-3.2	25	51.0	5	45.5	
3.3-24.0	24	49.0	6	54.5	
Gender					0.345
Male	34	69.4	6	54.5	
Female	15	30.6	5	45.5	
Age at recruitment (*M*) (*SD*)	(59.7)	(10.5)	(64.0)	(11.5)	0.317
35.7-61.0	26	53.1	4	36.4	
61.1-75.5	23	46.9	7	63.6	
Total	49	100.0	11	100.0	

Table S2 – Comparison between the randomly selected patients who accepted and refused to participate in the study according to the distribution of some clinical and demographic characteristics.

Legenda – N/%: absolute/relative frequency; M: mean; SD: standard deviation; P-value: probability level associated with the chi-squared test.

Clinical status	*HDg*		*IARAg*		*P-value*
	N	%	*N*	%	
Alive	21	77.8	16	72.7	0.724
Transplanted	2	7.4	2	9.1	
Deceased	4	14.8	3	13.6	
Dropout	0	0.0	1	4.5	
Total	27	100.0	22	100.0	-

Table S2 – Clinical status at the end of follow-up of patients who participated in the study according to the treatment group (IARA vs control).
Legenda – N/%: absolute/relative frequency; M: mean; SD: standard deviation; P-value: probability level associated with the chi-squared test.
